# Reconfigurable photonic crystals enabled by pressure-responsive shape-memory polymers

**DOI:** 10.1038/ncomms8416

**Published:** 2015-06-15

**Authors:** Yin Fang, Yongliang Ni, Sin-Yen Leo, Curtis Taylor, Vito Basile, Peng Jiang

**Affiliations:** 1 Department of Chemical Engineering, University of Florida, Museum Road, Gainesville, Florida 32611, USA; 2 Department of Mechanical and Aerospace Engineering, University of Florida, Gainesville, Florida 32611, USA; 3 ITIA-CNR, Industrial Technologies and Automation Institute, National Council of Research, Via Bassini, 15, 20133 Milano, Italy

## Abstract

Smart shape-memory polymers can memorize and recover their permanent shape in response to an external stimulus (for example, heat). They have been extensively exploited for a wide spectrum of applications ranging from biomedical devices to aerospace morphing structures. However, most of the existing shape-memory polymers are thermoresponsive and their performance is hindered by heat-demanding programming and recovery steps. Although pressure is an easily adjustable process variable such as temperature, pressure-responsive shape-memory polymers are largely unexplored. Here we report a series of shape-memory polymers that enable unusual ‘cold' programming and instantaneous shape recovery triggered by applying a contact pressure at ambient conditions. Moreover, the interdisciplinary integration of scientific principles drawn from two disparate fields—the fast-growing photonic crystal and shape-memory polymer technologies—enables fabrication of reconfigurable photonic crystals and simultaneously provides a simple and sensitive optical technique for investigating the intriguing shape-memory effects at nanoscale.

## Abstract (web summary)

Smart shape-memory polymers based on pressure stimuli have potential biomedical and aerospace applications but are largely unexplored. Here, Fang *et al.* present a reconfigurable photonic crystal that is reprogrammed at ambient conditions by a pressure-responsive shape-memory polymer.

## Graphical abstract

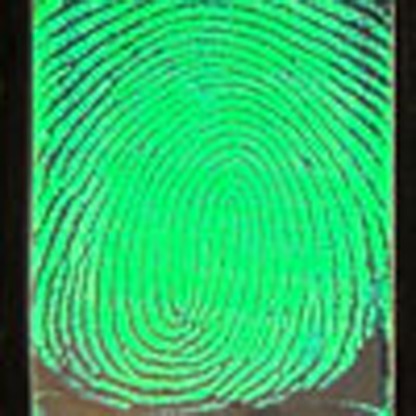

Shape-memory (SM) in traditional SM polymers (SMPs) is typically achieved in three steps including programming, storage and recovery^1^,^2^,^3^,^4^,^5^,^6^. Programming involves deforming a bulk SMP sample from its permanent shape to a temporary configuration. This ‘hot' process is usually done above a specific transition temperature (*T*_trans_), such as the polymer glass transition temperature (*T*_g_), to leverage the compliant properties of SMPs at high temperature. Once the sample is deformed, it is cooled below *T*_trans_ to ‘freeze' in the temporary shape. Recovery occurs when the sample is reheated to the vicinity of *T*_trans_, which increases polymer chain mobility and allows the SMP to return to its permanent shape via entropy elasticity^1^. Unfortunately, heat-demanding ‘hot' programming is generally used by almost every class of existing SMPs. By contrast, SMPs that can be ‘cold' programmed (that is, deformed to a temporary shape at or below room temperature), which could greatly enhance the processability to accommodate broader application requirements (for example, room-temperature operations for the entire SM cycle), are rare^7^,^8^. In addition, most of the current SMP applications focus on leveraging the macroscopic SM effects, where the deformation length scale is large (on the order of centimetres). However, an intriguing potential for all SMPs, largely unexplored, is their ability to memorize and change shape at nanoscale^9^,^10^,^11^,^12^,^13^,^14^.

Here, we report a new type of SMP that enables ‘cold' programming and instantaneous, nanoscopic shape recovery at ambient conditions. No heat is needed for both SM programming and recovery steps. Instead the ‘cold' programming is induced by capillary pressure produced by water evaporation from the SMP membranes. Contrary to traditional thermoresponsive SMPs, the SM recovery of the new SMPs can be achieved by applying a small contact pressure or drying the films out of solvents with low surface tension (for example, ethanol). Interestingly, by combining this new type of SMP with macroporous photonic crystals, we demonstrate the fabrication of reconfigurable/rewritable photonic crystal micropatterns by a simple direct print approach. Furthermore, *in-situ* nanoindentation tests reveal that the counterintuitive pressure-induced SM recovery is caused by an adhesive pull-off force between the contact substrate and the SMP membrane.

## Results

### Preparation and characterization of new SMPs

**Figure 1:**
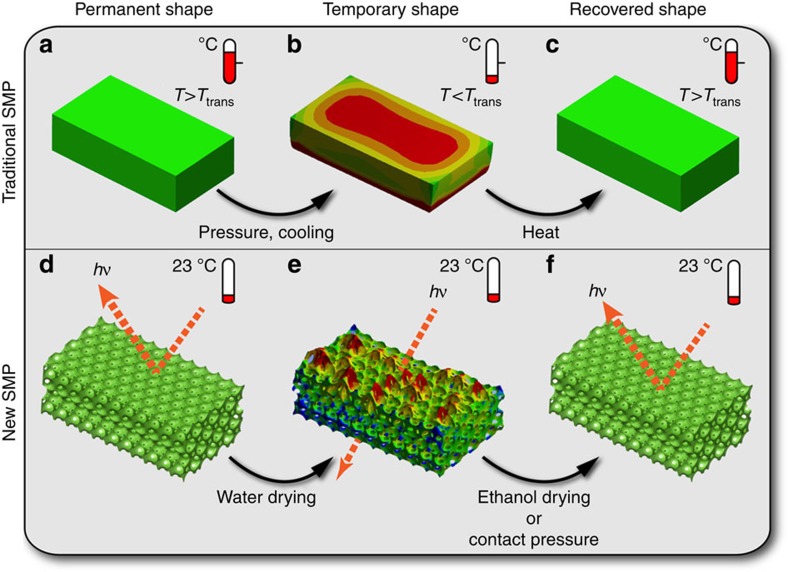
Schematic illustration compares different SM effects. (**a**) A traditional thermoresponsive SMP sample can be deformed into a temporary shape by a ‘hot' programming process (at a temperature above a specific *T*_trans_). (**b**) The temperature is cooled below *T*_trans_ to ‘freeze' in the temporary shape. (**c**) The recovery of the permanent shape can be triggered by applying heat (*T*>*T*_trans_) to the strained SMP sample. (**d**) A thin (a few micrometres thick) macroporous SMP photonic crystal with 3D ordered macropores (permanent shape) shows strong Bragg diffraction of visible light. (**e**) The ordered macropores can be deformed to a disordered structure (temporary shape) with no Bragg diffraction induced by an autonomous ‘cold' programming process (water drying) at ambient conditions. (**f**) The nanoscopic recovery of the permanent 3D photonic crystal structure can be stimulated by drying the sample out of ethanol or applying an external contact pressure.

**Figure 2:**
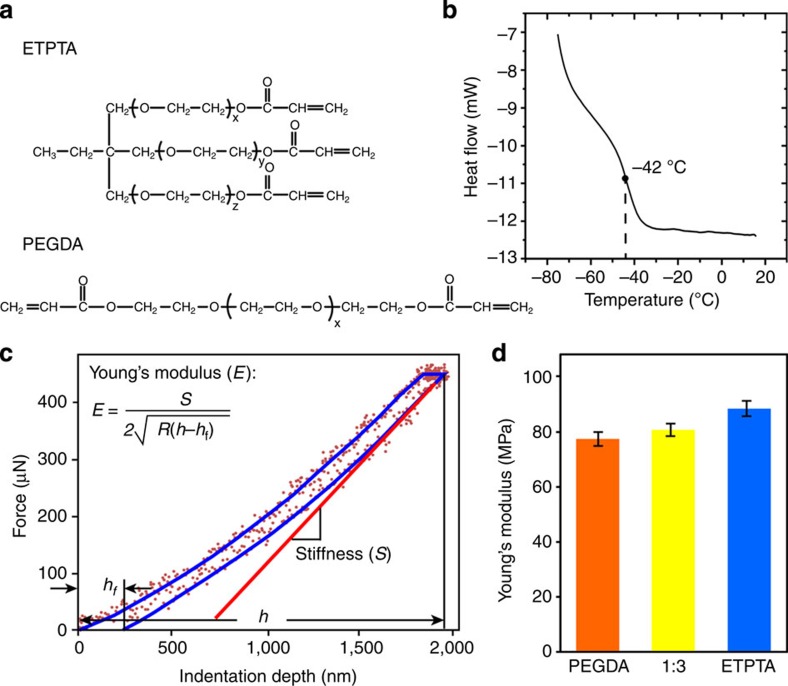
Structures and Properties of the new SMPs. (**a**) Molecular structures of ETPTA (*x*+*y*+*z*=20) and PEGDA (*x*=12) oligomers. (**b**) Differential scanning calorimetry plot of a macroporous ETPTA-co-PEGDA copolymer with 1:3 ratio. (**c**) Typical force–depth indentation curve used to measure elastic properties of SMP. (**d**) Average Young's moduli for PEGDA, ETPTA-co-PEGDA copolymer with 1:3 ratio and ETPTA polymers.

The new pressure-responsive SMPs were discovered in the fabrication of macroporous polymer photonic crystal membranes^15^,^16^. Photonic crystals are periodic dielectric structures with a forbidden photonic band gap (PBG) for electromagnetic waves^17^,^18^,^19^. They may hold the key to continued progress towards all-optical integrated circuits and high-speed optical computing^19^. Figure 1 compares the significant differences in SM effects between traditional thermoresponsive SMPs (Fig. 1a–c) and the new stimuli-responsive SMPs (Fig. 1d–f). The new SMPs are photocured copolymers of ethoxylated (20) trimethylolpropane triacrylate (ETPTA, *T*_g_∼−40 °C provided by the vendor) and polyethylene glycol (600) diacrylate (PEGDA, *T*_g_∼−42 °C) oligomers (see molecular structures in Fig. 2a) with varying volumetric ratios from 1:1 to 1:6. As the ETPTA-co-PEGDA copolymer with 1:3 ratio showed the optimal SM behaviours, this recipe was adopted throughout the current work if not explicitly stated otherwise. A single *T*_g_ of∼−42 °C measured by differential scanning calorimetry (Fig. 2b) of an SMP sample indicates the cross-linked copolymer is a homogeneous mixture of the two components. The Young's moduli of the pure ETPTA and PEGDA polymers and their 1:3 copolymer were characterized by *in-situ* nanoindentation tests (see the typical force-depth indentation curve in Fig. 2c). The results in Fig. 2d show that the average Young's moduli for all samples are about 80 MPa. The tensile strength of the 1:3 copolymer membrane measured by using a conventional tensile tester is ∼7.5 MPa and the yielding strain is ∼0.19 (Supplementary Fig. 1), indicating the copolymer is quite elastic at room temperature. The bulk Young's modulus is caculated to be ∼39 MPa, which is lower than the microscopic moduli measured by nanoindentation.

### Unusual ‘cold' programming caused by water evaporation

**Figure 3:**
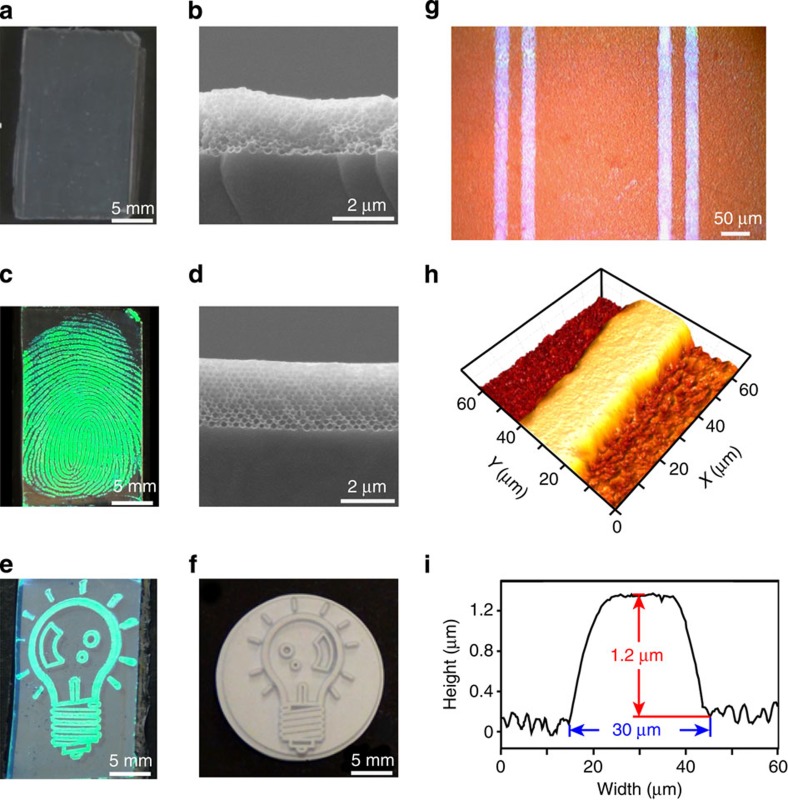
Arbitrary photonic crystal patterns printed on the new SMP membranes. (**a**) Photograph of a translucent macroporous SMP film with disordered macropores. (**b**) Cross-sectional SEM image of the macroporous sample in **a** with deformed macropores. (**c**) Photograph of a green-coloured fingerprint pressed on the sample in **a**. (**d**) Cross-sectional SEM image of an iridescent region in **c** with 3D ordered macropores (300 nm diameter). (**e**) Photograph of an iridescent ‘light bulb' pattern printed on a translucent macroporous SMP membrane templated from 300 nm silica microspheres. (**f**) Photograph of the rubber stamp used in generating the ‘light bulb' pattern in **e**. (**g**) Optical microscopic image of micropatterned pairs of double lines on a macroporous SMP membrane. (**h**,**i**) 3-D AFM image and the height profile of a section of a line in **g**.

Macroporous ETPTA-co-PEGDA membranes were fabricated by using self-assembled, three-dimensional (3D) highly ordered silica colloidal crystals as structural templates^15^. After removing the templating silica microspheres by a hydrofluoric acid wash, the resulting macroporous copolymer film immersed in water exhibited iridescent colours caused by Bragg diffraction of visible light from the periodic arrays of polymer macropores. This confirmed the maintenance of the 3D ordered structure of the original silica colloidal crystal. Surprisingly, the shining colours of the macroporous photonic crystal disappeared when the membrane was dried out of water and it became translucent with a pale white appearance (Fig. 3a, also see Supplementary Video 1). This suggests that the 3D periodic structure was lost when water evaporated from the ordered macropores. The cross-sectional scanning electron microscope (SEM) image in Fig. 3b confirms this conjecture, as no ordering of the deformed macropores is shown. Therefore, the new elastic copolymers enable an autonomous ‘cold' programming process—the deformation from a 3D ordered permanent structure to a disordered temporary structure can be achieved at ambient conditions by evaporating water from the templated macropores. This is in sharp contrast to traditional SMPs that need to be heated above *T*_trans_, then deformed to a temporary shape^1^.

### Pressure-induced SM recovery

Even more interesting, the recovery of the permanent photonic crystal structure can be triggered at room temperature simply by applying a small contact pressure on the macroporous membranes with collapsed macropores. As illustrated by the fingerprinting process in Supplementary Video 2, an iridescent green-coloured fingerprint (Fig. 3c) immediately appeared on the translucent macroporous copolymer membrane templated from 300 nm silica microspheres. The cross-sectional SEM image in Fig. 3d shows a fingerprinted region with the vivid green colour and the recovered 3D highly ordered macroporous structure is evident. The difference in the surface microstructures between the fingerprint valleys and ridges was characterized by atomic force microscopy (AFM, Supplementary Fig. 2). The surface of the raised fingerprint ridges is apparently much smoother than that of the valleys as confirmed by the magnified AFM images and the surface roughness analysis (Supplementary Table 1). The raising height of the fingerprint ridges above the valleys is estimated to be ∼1.5 μm by the AFM depth profile. The gradual transition from a disordered macroporous array in a fingerprint valley to a 3D highly ordered structure in a fingerprint ridge is shown by the SEM image in Supplementary Fig. 3.

To avoid possible body-temperature effects on the macropore recovery in the above fingerprinting process, as well as to verify the feasibility of a new printing-based technology for fabricating arbitrary photonic crystal patterns, we printed a ‘light bulb' relief pattern on a rubber stamp (Fig. 3f) onto a translucent SMP copolymer membrane with collapsed 300 nm macropores at room temperature. The final iridescent imprint (Fig. 3e) is a faithful replica of the original relief pattern. Furthermore, standard microfabrication technologies were used in making microscopic patterns on silicon wafers, which were used to imprint the micropatterns on SMP copolymer membranes. Figure 3g shows an optical microscope image of printed pairs of parallel lines with ∼30 μm width. The raising up of the smoother line patterns from the rough macroporous surface is clearly presented by the AFM image (Fig. 3h) and the corresponding depth profile (Fig. 3i). Although various technologies for fabricating tunable photonic crystals have been demonstrated using elastic materials (for exmple, elastomers and gels), the temporarily deformed photonic microstructures cannot be memorized^18^,^20^,^21^,^22^,^23^. Immediately, they return to the original crystalline lattices once the external stress is released. By contrast, the printed photonic crystal patterns on the new pressure-responsive SMPs are stable over long periods of time. The colourful fingerprints (for example, Fig. 3c) stored at ambient conditions have maintained their vivid colours and clear patterns for more than 2 years. Most importantly, the imprinted photonic crystal patterns can be erased when the SMP membranes are reimmersed in water and then dried out of it (see Supplementary Video 3). New photonic microstructures can then be printed on the regenerated macroporous SMP membranes. This unique rewriting capability is critical for developing reconfigurable photonic crystals that can adapt various photonic functionalities to accommodate different applications^24^,^25^. This reconfigurability can dramatically reduce the complexity and fabrication cost of developing a large number of application-specific devices^26^.

### Capillary pressure-induced macropore collapse

Above we have shown that the new SMP copolymers enable room-temperature operations for the entire SM cycle (from an unusual ‘cold' programming process to a contact pressure-induced recovery step). We speculated that the ‘cold' programming process was induced by large capillary pressure created by water evaporation from the template macropores, which squeezed the elastic macropores into disordered arrays^27^. Similar macropore collapse was observed for macroporous polymer (for example, polysulfone) reverse osmosis membranes used for water purification^28^. Further insight into the macropore collapse can be gained by considering the capillary pressure (*P*_c_) in the Young–Laplace equation, *P*_c_*=2γ cosθ/r*, where *γ* is the liquid/vapour surface tension, *r* is the radius of the pores and *θ* is the contact angle of the liquid on the pore surface^27^. As the measured water contact angles on the copolymers were <20°, *cosθ* is thus close to 1. One direct evidence supporting this capillary pressure-induced macropore collapse mechanism is that the templated macroporous membrane retained its original 3D ordered structure and iridescent colours when dried out of ethanol (Supplementary Video 4), which has a smaller surface tension than that of water (22.39 versus 72.75 mN m^−1^ at 20 °C). The smaller *γ* led to a lower *P*_c_ that was not sufficient to squeeze the elastic macropores into disordered arrays. In addition to ethanol, a large variety of solvents with low surface tension (for example, acetone and toluene) can also trigger the same disorder-to-order transition.

**Figure 4:**
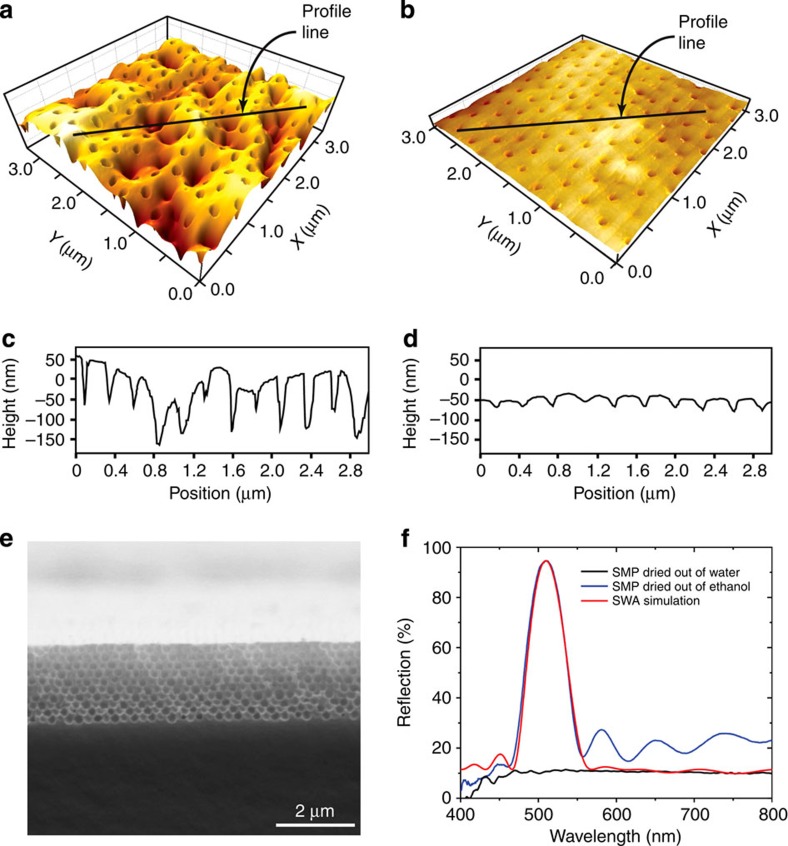
Difference in topography and PBGs. (**a**,**c**) 3D AFM image and the height profile scanned across the profile line for a water-dried SMP sample consisting of 280 nm macropores. (**b**,**d**) 3D AFM image and the height profile for the same sample dried out of ethanol. (**e**) Cross-sectional SEM image of the sample in **b**. (**f**) Normal-incidence optical reflection spectra compares the PBG properties of the macroporous samples dried out of water and ethanol. The scalar-wave approximation-simulated spectrum assuming a perfect macroporous crystalline lattice is also shown to compare with the experimental results.

Figure 4a–d compare the surface microstructures of a macroporous SMP copolymer membrane dried out of water (Fig. 4a,c) and ethanol (Fig. 4b,d), respectively. The rough, disordered macroporous array was fully recovered to a smooth and ordered structure triggered by ethanol evaporation (Fig. 4e). This disorder-to-order transition and the corresponding translucent-to-iridescent colour change can be characterized by measuring the normal-incidence optical reflection spectra. In Fig. 4f, the sample with disordered macropores (dried out of water) shows no apparent PBG peaks, whereas the ethanol-recovered sample with ordered macropores exhibits a distinct PBG peak with well-defined Fabry–Perot fringes, indicating the high crystalline quality of the solvent-activated macroporous photonic crystal^15^. Importantly, the experimental spectrum of the recovered photonic crystal matches well with the calculated spectrum using a scalar-wave approximation model^29^, which assumes a perfect macroporous crystalline lattice. This indicates that the temporarily deformed macropores were fully reopened to their permanent, 3D highly ordered structure triggered by ethanol evaporation.

### Critical contact pressure for SM recovery

**Figure 5:**
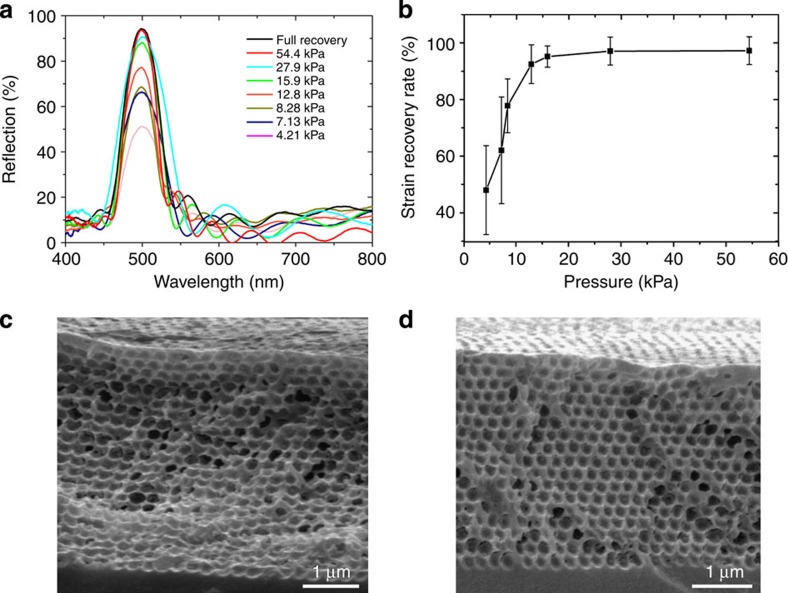
Pressure-dependent macropore recovery. (**a**) Normal-incidence optical reflection spectra obtained from a macroporous SMP membrane consisting of 280 nm macropores under different pressures. The same sample dried out of ethanol was labelled as ‘Full Recovery'. (**b**) Normalized absolute reflection amplitude of a recovered SMP sample was used as an indicator of the nanoscopic SMP strain recovery rate under different pressures. (**c**,**d**) Cross-sectional SEM images of a macroporous SMP membrane recovered by 7.13 and 27.9 kPa pressure, respectively.

By using the ethanol-activated sample as a fully recovered control, we can estimate the critical pressure that is needed to trigger the shape recovery and the nanoscopic strain recovery rate (*R*_r_) of the new SMPs through monitoring the PBG properties of the recovered samples under different pressures. Figure 5a shows that different reflection amplitudes of the PBG peaks resulted when various pressures were applied by putting varying weights on a small polydimethylsiloxane piece with a specific area on a macroporous copolymer membrane. Previous work has shown that the PBG optical density of a macroporous photonic crystal is nearly a linear function of its crystalline thickness^15^. We therefore normalized the absolute reflection amplitude (after spectrum baseline correction) of a pressure-recovered SMP sample to that of the ethanol-activated control sample as an indicator of *R*_r_ (Fig. 5b). A near-unity *R*_r_ was obtained when a 54.4-kPa pressure was applied, whereas a ∼50% recovery needed a pressure of 4.21 kPa. The cross-sectional SEM images in Fig. 5c,d, which correspond to the samples recovered by applying 7.13 and 27.9 kPa pressure, responsively, reveal that an intermediate pressure only induces the partial recovery of the top layers of the macroporous SMP photonic crystal, leading to the lower reflection amplitude compared with the fully recovered control sample.

### Pressure-induced SM recovery mechanisms

**Figure 6:**
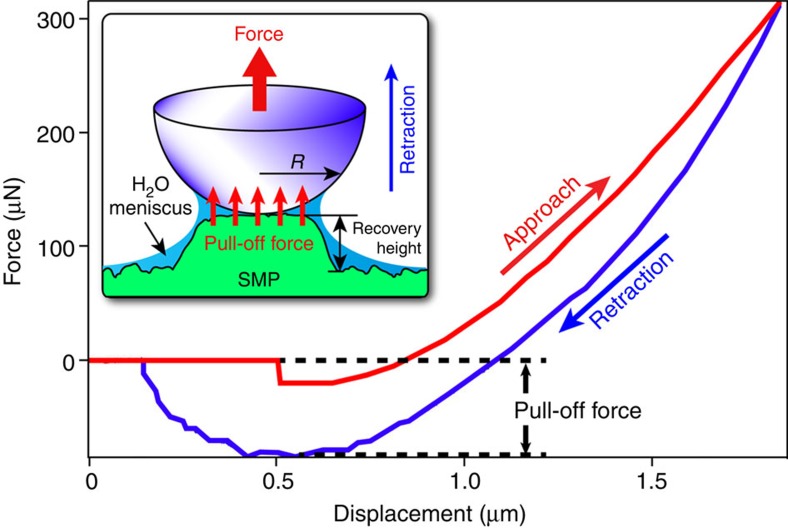
Macropore recovery induced by pull-off forces. A typical indentation force-displacement curve showing approach and retraction segments and pull-off force obtained on a macroporous SMP membrane. Inset shows diagram of macropore recovery during retraction of indenter due to pull-off force caused by van der Waals interactions and the capillary force induced by the capillary-condensed water meniscus layer between the indenter tip and the SMP membrane.

Similar to thermoresponsive SMPs, we believe the entropy elasticity^1^ is the energetic root cause for the SM effects exhibited by the new pressure-responsive SMPs. When photocured in the interstitial regions of the templating colloidal crystal, the cross-linked polymer chains were primarily in energetically favourable, stress-free configurations. The capillary pressure-induced ‘cold' programming squeezed the ordered macropores into disordered arrays with reduced thickness (see Fig. 3b,d), storing excess stresses in the deformed, temporarily configured polymer chains. The strained polymer networks have a strong tendency to recover back to their permanent, stress-free states. However, the observed pressure-induced macropore recovery is counterintuitive as we expect the applied pressure should further deform the collapsed macropores instead of popping them up. To elucidate this unusual shape-recovery mechanism, we conducted *in-situ* nanoindentation tests to characterize the forces in the approaching and retracting processes when a spherical sapphire tip indented the macroporous SMP membrane (Fig. 6). An apparent adhesive pull-off force, caused by the attractive van der Waals interactions and the capillary force induced by the capillary-condensed water meniscus layer between the indenter tip and the SMP membrane^30^, is evident in the retraction process. We believe this pull-off force causes the SM recovery of the collapsed macropores. A higher pressure leads to more conformal interactions between the molecules on the tip and the membrane, and thus a larger pull-off force. This could explain the pressure effects on the strain recovery rate (Fig. 5b) and the partial recovery of the top-layer macropores when an intermediate pressure was applied (Fig. 5c). One strong evidence supporting this pull-off mechanism is that the collapsed macropores did not recover back to their ordered structure when a pressure (up to∼350 kPa) was applied through compressed air instead of a contacting body or entity (for example, a finger or a rubber stamp), which could exert the pull-off force on the SMP macropores.

## Discussion

To conclude, we have developed a new type of stimuli-responsive SMP that differs greatly from existing SMPs, as it enables fast response and room-temperature operations for the entire SM cycle. The large capillary pressure generated by water evaporation from the templated macropores induces the unusual ‘cold' programming at room temperature. The instantaneous recovery of the temporarily deformed macropores to the permanent, 3D highly ordered photonic crystal structure can be triggered by applying a small contact pressure at ambient conditions. In addition to being pressure responsive, the disorder-to-order transition of the new SMPs can also be stimulated by drying the macroporous SMP membranes out of solvents with low surface tension, such as ethanol and toluene. Importantly, the easily perceived colour change from translucency to iridescence associated with the structural disorder-to-order transition enables a simple and quantitative optical technology for characterizing the intriguing SM effects at nanoscale. Simultaneously, the striking chromogenic effects induced by the recovery of the permanent 3D photonic crystal structure provide opportunities for a wide spectrum of applications ranging from reconfigurable photonic crystal devices to chromogenic pressure and chemical sensors to novel biometric and anti-counterfeiting materials.

## Methods

### Templated fabrication of macroporous SMP photonic crystal membranes

Monodispersed silica microspheres, with diameter ranging from 100 to 600 nm, were synthesized by the standard Stöber method. Silica particles were purified in 200-proof ethanol by multiple centrifugation and redispersion cycles. Next, they were self-assembled on a glass microslide by the convective self-assembly technology to form colloidal crystals^15^. By adjusting the particle volume fraction of the silica/ethanol suspension, the thickness of the colloidal crystal was controlled to 10–50 colloidal monolayers. The microslide with the silica colloidal crystal on its surface was covered by another microslide and a double-sided adhesive tape of ∼1 mm thick was used as a spacer in between the microslides. By using capillary force, the interstitial air in between the silica microspheres was replaced by viscous oligomer mixtures consisting of ETPTA (SR415, Sartomer, molecular weight 1,176 kDa, viscosity 225 cps at 25 °C, refractive index 1.470) and PEGDA (SR610, Sartomer, molecular weight 742 kDa, viscosity 90 cps at 25 °C, refractive index 1.468) oligomers with varying volumetric ratios from 1:1 to 1:6. Darocur 1173 (2-hydroxy-2-methyl-1-phenyl-1-propanone, BASF, 1 wt%) was added as the photoinitiator. The sample was transferred to a pulsed ultraviolet curing system (RC 742, Xenon) and the oligomer mixture was rapidly polymerized by exposure to ultraviolet radiation for 4 s. The polymerized film was soaked in a 1 vol% hydrofluoric acid aqueous solution for 4 h and then rinsed with deionized water. The resulting self-standing macroporous polymer membrane showed pale iridescent colours when immersed in water and observed at large viewing angles (>45°) due to the small refractive index contrast between the copolymer (∼1.47) and water (1.33).

### Printing photonic crystal patterns on macroporous SMP membranes

Using Kimwipes, the free-standing macroporous SMP membrane was dried and the diffractive colours of the film were lost during water evaporation (see Supplementary Video 1). Strikingly iridescent photonic crystal patterns, whose colours are determined by the size of the templating silica microspheres, can be printed on the translucent SMP membranes by using various substances with relief patterns, such as fingers or rubber stamps (see Fig. 3c,e). To generate microscopic photonic crystal patterns, standard photolithography and chlorine reactive ion etching were performed in a class 100 cleanroom, to fabricate micropatterns (for example, parallel lines in Fig. 3g) on a silicon wafer. The hard silicon mould was then placed on an SMP membrane and a typical fingerprinting pressure was applied on the mould to transfer the micropatterns. To evaluate the pressure effects on the macroporous SMP strain-recovery rate, we placed different weights (43, 73, 85, 130, 162, 285 and 555 g) on a small polydimethylsiloxane square (1 cm × 1 cm, Sylgard 184, cured at 75 °C for 2 h), to generate various pressures on a macroporous copolymer membrane.

### Sample characterization

SEM imaging was carried out on a FEI XL-40 FEG-SEM. A thin layer of gold was sputtered onto the samples before imaging. Amplitude-modulation AFM was performed using a MFP-3D AFM (Asylum Research, Inc.) with a Nanosensor PPP-NCHR probe (tip radius <10 nm), to characterize the topography and surface roughness of macroporous SMP membranes. *In-situ* nanoindentation tests were performed with an MFP-3D NanoIndenter (Asylum Research, Inc.) using a spherical sapphire indenter (tip radius ∼125 μm). Such configuration of the instrument has a force and displacement resolution <3 μN and 1 nm, respectively. Detailed surface roughness analysis and calculation of Young's modulus by nanoindentation are discussed in Supplementary Information. An Instron model 1122 load frame upgraded with an MTS ReNew system and equipped with a 500-g load cell at a cross-head speed of 0.5 mm min^−1^ was used in testing the tensile strength of the SMP membranes. Differential scanning calorimetric measurements were carried out from −80 to 18 °C at a heating rate of 10 °C min^−1^ using a Seiko DSC 6200 instrument and an empty pan as reference. Normal-incidence optical reflection spectra were obtained using an Ocean Optics HR4000 high-resolution fibre optic visible-near infrared spectrometer with a reflection probe (R-400-7-SR) and a tungsten halogen light source (LS-1). Absolute reflectivity was obtained as the ratio of the sample spectrum and a reference spectrum, which was the optical density obtained from an aluminum-sputtered (1,000 nm thickness) silicon wafer.

### Scalar wave approximation optical modelling

The scalar wave theory developed for periodic dielectric structures^29^ was implemented to calculate the normal-incidence optical reflection spectra from macroporous SMP photonic crystals. In this theory, Maxwell's equations are solved for a periodic dielectric assuming that one may neglect diffraction from all but one set of crystalline planes (for example, the (111) planes in our case). The scalar-wave approximation calculation contains no adjustable parameters, as the size of the macropores and the crystal thickness were independently determined from SEM characterization and the refractive index of the SMP copolymer is known.

## Additional information

**How to cite this article**: Fang, Y. *et al.* Reconfigurable photonic crystals enabled by pressure-responsive shape-memory polymers. *Nat. Commun.* 6:7416 doi: 10.1038/ncomms8416 (2015).

## Supplementary Material

Supplementary Information Supplementary Figures 1-3, Supplementary Tables 1, Supplementary Discussion and Supplementary References.

Supplementary Movie 1 A free-standing macroporous SMP membrane templated from 300 nm silica microspheres dried out of water.

Supplementary Movie 2 Fingerprinting on a translucent macroporous SMP membrane templated from 300 nm silica microspheres.

Supplementary Movie 3 Regenerating a new fingerprint on a macroporous SMP membrane by erasing an old fingerprint by consecutively drying the sample out of ethanol and water.

Supplementary Movie 4 A translucent macroporous SMP membrane templated from 300 nm silica microspheres dried out of ethanol.
