# Supplementary material for: Reconfigurable photonic crystals enabled by pressure-responsive shape-memory polymers
Source: Nat Commun. 2015 Jun 15;6:7416. doi: 10.1038/ncomms8416 (PMC4490579; doi:10.1038/ncomms8416)
Supplement: Supplementary Information — Supplementary Figures 1-3, Supplementary Tables 1, Supplementary Discussion and Supplementary References. [file ncomms8416-s1.pdf]

Supplementary Information

1. Supplementary Figures

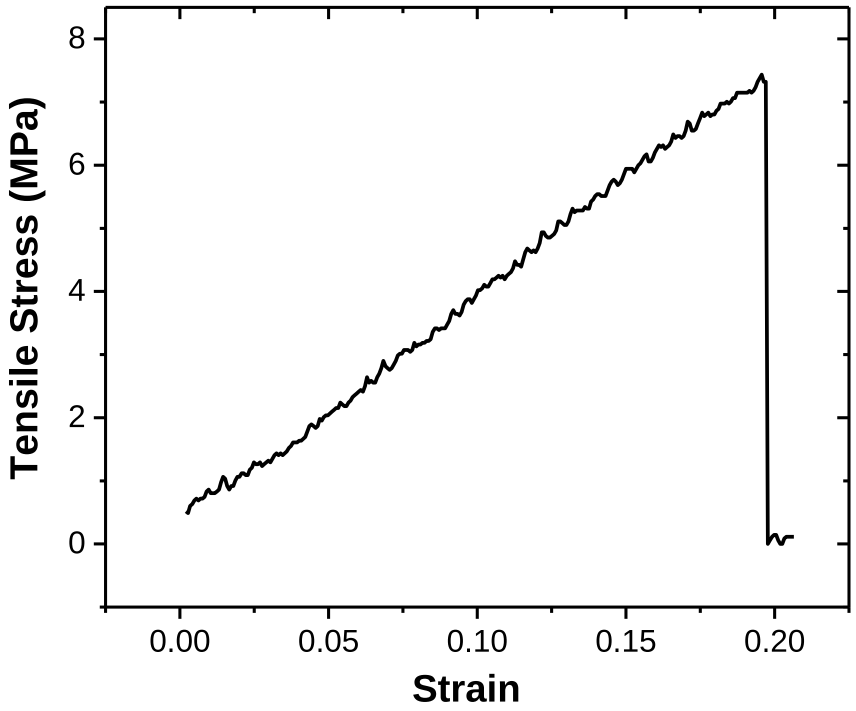

Supplementary Figure 1 Typical tensile stress versus strain curve for an ETPTA-co-PEGDA copolymer membrane with 1:3 ratio.

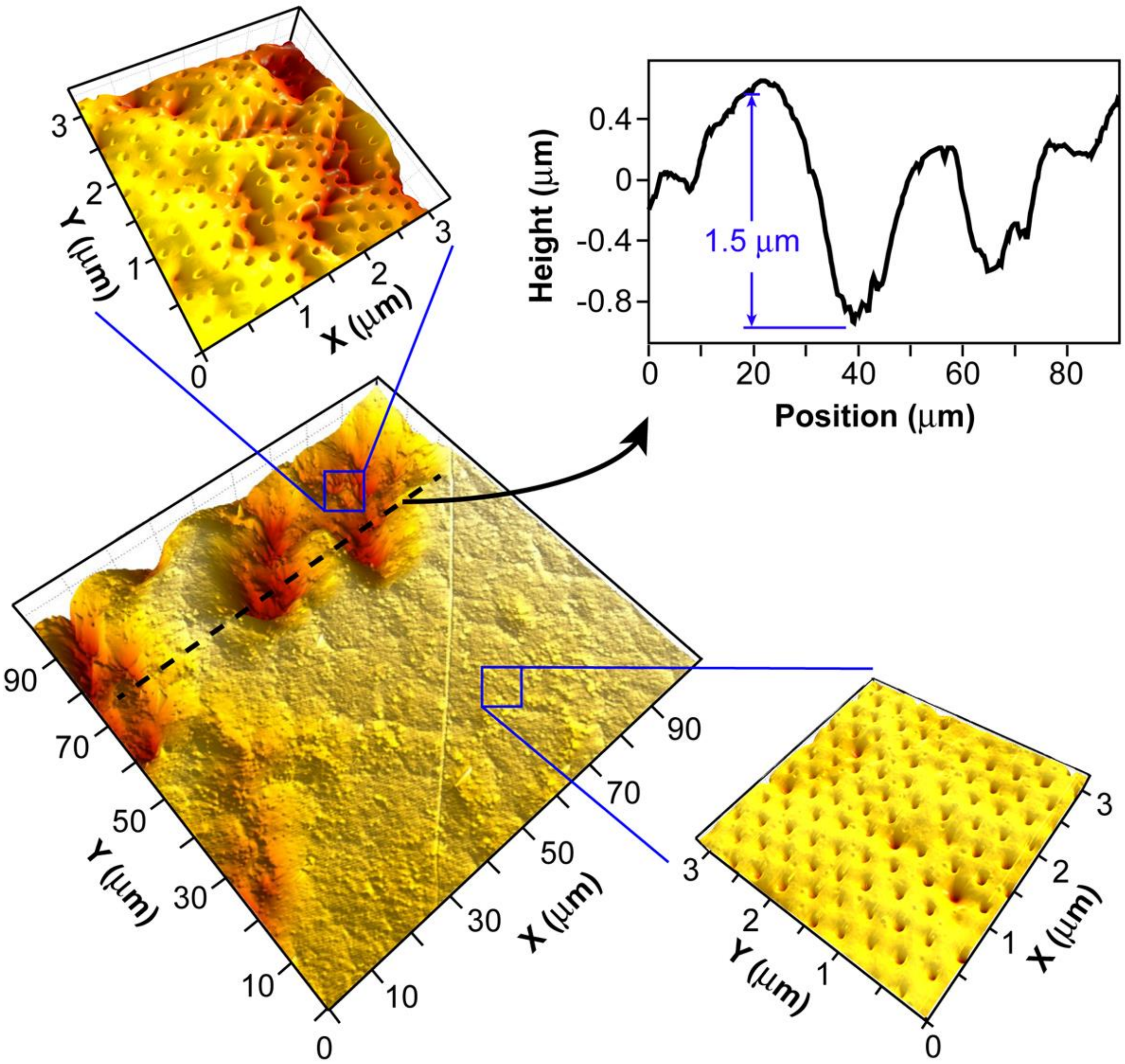

Supplementary Figure 2 3-D AFM images of a fingerprinted SMP sample with cross-sectional profiles. Inset: (Left) The $3 \times 3\ \mu m^2$ AFM image inside the valley region of the $90 \times 90\ \mu m^2$ fingerprint sample surface. (Right) The $3 \times 3\ \mu m^2$ AFM image inside the raised ridge region of the $90 \times 90\ \mu m^2$ fingerprint sample surface. (Middle) The cross-sectional profile of the $90 \times 90\ \mu m^2$ fingerprint sample surface.

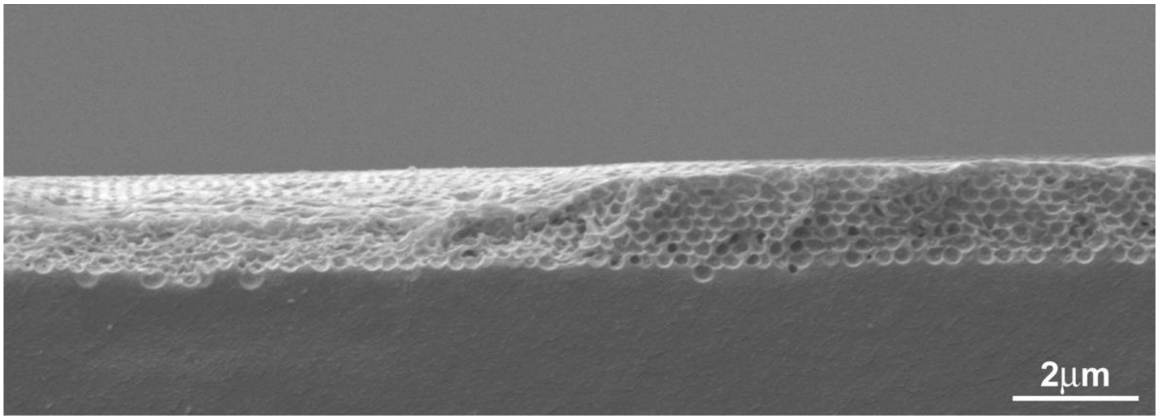

Supplementary Figure 3 Cross-sectional SEM image shows the transition from the disordered fingerprint valley region to the 3-D ordered fingerprint ridge region.

2. Supplementary Tables

Supplementary Table 1 Roughness of 10 × 10 μm^2 AFM scan size of SMP surface.

Sample	3D Areal Roughness		Linear Profile Roughness	
	AA Roughness S_a (nm)	RMS Roughness S_q (nm)	AA Roughness R_a (nm)	RMS Roughness R_q (nm)
**Water-dried**	46.5 ± 7.1	59.4 ± 9.9	34.6 ± 6.3	41.7 ± 7.8
**Ethanol-dried**	8.7 ± 2.5	11.9 ± 4.3	6.3 ± 1.2	7.7 ± 1.3
**FP valley**	64.2 ± 21.6	84.9 ± 26.7	47.4 ± 15	56.5 ± 17.6
**FP ridge**	11.8 ± 2.9	16.4 ± 4.4	8.7 ± 2.1	10.9 ± 2.6

3. Supplementary Discussion

Young's moduli of the new SMPs determined by nanoindentation. Nanoindentation can be used to determine the Young's modulus of a broad range of materials. By pressing an indenter into a material with a predefined depth or force, a force-displacement curve can be generated. By fitting the curve with an appropriate contact mechanics model (i.e. Oliver-Pharr model), material properties such as Young's modulus and hardness can be extracted. Indentation tests were

performed with a MFP-3D NanoIndenter (Asylum Research, Inc.) using a spherical sapphire indenter (tip radius ~125 μm). Such configuration of the instrument has a force and displacement resolution less than 3 μN and 1 nm, respectively. Due to the comparatively large contact radius of the spherical tip, there was no need to perform a tip area calibration according to Oliver and Pharr[1]. Tip geometry was directly measured by optical microscope. A force-controlled trapezoidal load function with a 5-2-2 seconds segments corresponding to loading-hold-unloading times was applied to all indentations. Three forces (100 μN, 200 μN, and 300 μN) were chosen to compare the Young's modulus of different indentation forces/depths. With each force, ten impressions were indented on each sample with an inter-distance of 200 μm, which is ten times over the average residual impression size. All indentations were triggered by 7.5 μN force, corresponding to ~2 nm deflection in the indenter spring. Overall, 30 indents were made on each sample. All indents were made at room temperature (23 °C) and the system was allowed to reach thermal equilibrium for 30 minutes prior to indentation to minimize the thermal drift effect.

To calculate the Young's modulus, the force-displacement curves obtained from indentation experiments were fitted with the Oliver-Pharr model[1] in 80% − 20% portion of the unloading curves. The calculations were conducted using IGOR Pro analysis software routine (WaveMetrics Inc.). The fitting curves are in power law function form:

$$P = \alpha(h - h_f)^m \quad (1)$$

where α and m are power law fitting constants. h_f is the final depth of the contact impression after unloading.

The contact depth is defined as the difference of the maximum indentation depth and the sink-in depth

$$h_c = h_{max} - \epsilon \frac{P_{max}}{S} \quad (2)$$

where ϵ is the indenter geometry parameter and S is the measured unloading stiffness, which is defined as

$$S = \beta \frac{2}{\sqrt{\pi}} E_{eff} \sqrt{A_c} \quad (3)$$

where β is a dimensionless parameter used to account for deviations in stiffness caused by lack of axial symmetry, A_c is the projected contact area of the indenter with repect to contact depth h_c,

$$A_c = -\pi h_c^2 + 2\pi R h_c \quad (4)$$

and E_{eff} is the effective (reduced) Young's modulus defined by

$$\frac{1}{E_{eff}} = \frac{1 - \upsilon_s^2}{E_s} + \frac{1 - \upsilon_i^2}{E_i} \quad (5)$$

where E_s , ν_s and E_i , ν_i are Young's modulus and Poisson's ratio of sample and indenter, respectively. R is the indenter tip radius.

According to the Oliver-Pharr method[1] and the manufacturer's specifications, several parameters used in our case were set as $\epsilon = 0.75, \beta = 1.05, \nu_s = 0.5, \nu_i = 0.29, and\ E_i = 350\ GPa.$

AFM surface microstructure characterization and roughness analysis. Amplitude-modulation atomic force microscopy (Asylum Research, Inc.) was used to characterize the topography of the macroporous SMP membranes. All AM-AFM imaging was performed using the MFP-3D AFM with a Nanosensor PPP-NCHR probe (tip radius < 10 nm). A total of 5 images were scanned in different locations on each sample with an average interval larger than 100 μm. All the 3×3 μm^2, 10×10 μm^2 and 90×90 μm^2 images were scanned with a data collection density of 512×512 pixels per image. For all images presented, the trace and retrace images of the topography matched excellently, ensuring the absence of image artifacts and the

accuracy of the data collected. Both the sample preparation and imaging were performed at room temperature (~ 23 °C) and relative humidity ~ 50%.

All the surface topographic images and the surface roughness were generated and calculated in the commercial software package Scanning Probe Imaging Processor (SPIP). A 1st order plane correction was performed to compensate for surface tilt. Cross-sectional profiles were measured from AFM imaging data to provide quantitative information, such as feature heights and lengths. The AFM images and the height profile scanned across the dashed line shown in Supplementary Fig. 2 illustrate the difference in the surface microstructures between the fingerprint valleys and ridges printed on a macroporous SMP membrane with 300 nm macropores.

The surface roughness was determined by both the arithmetic average (AA) roughness R_a and the root mean square (RMS) roughness R_q using AFM topographic images. Each average roughness datum was calculated from 15 roughness values – 3 samples with 5 different locations on each. Two different approaches were implemented to characterize the difference between the whole area (including pores) roughness and only non-porous area roughness:

(i) 3D areal roughness: This method includes whole surface area and porous features.

(ii) Linear profile roughness: This method extracts out several lines without porous features.

The roughness values were calculated according to the ASME B46.1. The formulas used in calculating the roughness are:

$$R_a = \frac{1}{n}\sum_{i=1}^{n}|y_i| \quad (5)$$

$$R_q = \sqrt{\frac{1}{n}\sum_{i=1}^{n} y_i^2} \quad (6)$$

$$S_a = \frac{1}{mn}\sum_{k=1}^{m}\sum_{l=1}^{n}|z(x_k, y_l)| \quad (7)$$

$$S_q = \sqrt{\frac{1}{mn}\sum_{k=1}^{m}\sum_{l=1}^{n}|z(x_k, y_l)|^2} \quad (8)$$

For our case, $\mathrm{m} = \mathrm{n} = 512$. Supplementary Table 1 summarizes the surface roughness results obtained from macroporous SMP membranes dried out of water and ethanol, and the fingerprint (FP) valley and ridge regions.

4. Supplementary References

1. Oliver, W. C. & Pharr, G. M. Measurement of hardness and elastic modulus by instrumented indentation: Advances in understanding and refinements to methodology. *J. Mater. Res.* **19**, 3-20 (2004).
